# Effect of nutritional recovery with soybean flour diet on body composition, energy balance and serum leptin concentration in adult rats

**DOI:** 10.1186/1743-7075-6-34

**Published:** 2009-08-24

**Authors:** Loanda Maria G Cheim, Elisângela A Oliveira, Vanessa C Arantes, Roberto V Veloso, Marise Auxiliadora B Reis, Maria Helena G Gomes-da-Silva, Everardo M Carneiro, Antonio C Boschero, Márcia Q Latorraca

**Affiliations:** 1 Departamento de Alimentos e Nutrição, Faculdade de Nutrição, Universidade Federal de Mato Grosso, Cuiabá, Mato Grosso, Brasil; 2 Secretaria de Estado de Saúde, Cuiabá, Mato Grosso, Brasil; 3 Departamento de Ciências Básicas em Saúde, Faculdade de Ciências Médicas, Universidade Federal de Mato Grosso, Cuiabá, Mato Grosso, Brasil; 4 Departamento de Fisiologia e Biofísica, Universidade Estadual de Campinas, Campinas, São Paulo, Brasil

## Abstract

**Background:**

Malnutrition in early life is associated with obesity in adulthood and soybean products may have a beneficial effect on its prevention and treatment. This study evaluated body composition, serum leptin and energy balance in adult rats subjected to protein restriction during the intrauterine stage and lactation and recovering on a soybean flour diet.

**Methods:**

Five groups of the Wistar strain of albino rats were used: CC, offspring born to and suckled by mothers fed a control diet and fed the same diet after weaning; CS, offspring born to and suckled by mothers fed a control diet and fed a soybean diet with 17% protein after weaning; LL, offspring of mothers fed a low protein diet and fed the same diet after weaning; LC, offspring of mothers fed a low protein diet, but fed a control diet after weaning; LS, offspring of mothers fed a low protein diet, but fed a soybean diet with 17% protein after weaning. Food intake, body, perirenal and retroperitoneal adipose tissue were measured in grams. Leptin was quantified using the Enzyme Linked Immuno Sorbent Assay (ELISA) and insulin by radioimmunoassay (RIA). Carcass composition was determined by chemical methods and energy expenditure was calculated by the difference between energy intake and carcass energy gain. Data were tested by analysis of variance (ANOVA).

**Results:**

The LC and LS groups had higher energetic intake concerning body weight, lower energy expenditure, proportion of fat carcass and fat pads than CC and CS groups. The LS group showed reduced body weight gain and lower energy efficiency, which was reflected in less energy gain as protein and the proportion of carcass protein, and lower energy gain as lipid than in the LC groups, although both groups had eaten the same amount of diet and showed equal energy expenditure. Serum leptin did not differ among groups and was unrelated to food or energy intake and energy expenditure. Serum insulin was higher in the LS than in the LC group.

**Conclusion:**

Protein restriction during intrauterine life and lactation periods did not provoke obesity in adulthood. Nutritional recovery with soybean diet decreased the body weight at the expense of lower energy efficiency with repercussion on lean mass.

## Introduction

Currently, around 600 million people worldwide suffer major nutritional problems including energy deficits and starvation, whilst approximately 300 million people have chronic problems such as energy surpluses and obesity and, in some cases, these problems co-exist [1,2]. The increase in obesity and the reasons why some individuals appear to gain weight so easily is a much-debated topic [3].

Epidemiological studies show a higher incidence of obesity among men whose mothers experienced food deprivation in pregnancy during the Dutch famine in the Second World War [4]. In addition, central adiposity and other features related to a metabolic syndrome in adults born underweight were also documented [5,6]. These findings suggest that malnutrition in early life could promote obesity in adulthood.

Body weight, food intake and metabolism are regulated by the hypothalamus, that processes central and peripheral signals. Within the hypothalamus, neurons residing in the ARC (arcuate nucleus) – PVN (paraventricular) – PF/LH (perifornical/lateral hypothalamus) axis communicate with each other and are subjected to the influence of several peripheral factors, including leptin [7]. Leptin is a hormone mainly produced by fat cells in which plasma levels are a function of fat mass [8,9].

It has been suggested that programming of leptin concentrations by early diet may be one mechanism that links early nutrition with later obesity [10]. Protein restriction is associated to increase in body fat, serum leptin concentrations and food intake, which suggests a state of leptin resistance [11]. When rats are undernourished during the first two weeks of pregnancy and re-fed during the third week, the male offspring develop hyperphagia and obesity in adulthood if maintained either on a high fat diet or on a standard low fat and high carbohydrate diet [12-15]. Protein and energy restriction during lactation increase expression of leptin receptors in the pituitary and maintain normal serum leptin concentrations in adult life [16]. Thus, poor nutrition in early life and the type of diet utilized during the recovery phase appear to be critical in the determination of obesity in adulthood.

Considerable energy has been invested in finding new therapeutic approaches to overcome obesity. Studies have shown that the consumption of soybean reduces the body weight and adiposity [17,18]. These effects were attributed either to soy protein, isoflavones and/or others components (saponins, fiber, etc.) that act together or separately. Soybean did not reduce food intake, but increased the activity of uncoupling protein 1 (UCP-1) in brown adipose tissue, enhanced lipolysis and inhibited the lipogenesis in the white adipose tissue [19-23]. In addition, isoflavones reduce serum leptin concentrations in male obese rats, because it diminishes the adiposity [21].

This study was undertaken to evaluate body composition, serum leptin concentration and energy balance in adult rats which had been subjected to protein restriction during intrauterine life and lactation and had recovered on whole soybean flour diet.

## Materials and methods

### Animals and diets

Experimental procedures followed the COBEA guidelines (Brazilian Committee on Experimental Animal Care) adapted by the Federal University of Mato Grosso. Virgin female Wistar rats (85–90 d old) were obtained from the University's own breeding colony. Paring was performed by housing males with females overnight, and pregnancy was confirmed by the examination of vaginal smears for the presence of sperm. Pregnant females were separated at random and maintained from the first day of pregnancy until the end of lactation on isocaloric diets containing 6% protein (LP diet) or 17% protein (C diet). Spontaneous delivery took place on day 22 of pregnancy after which, at 3 d of age, large litters were reduced to eight pups, thus ensuring a standard litter size per mother. At weaning (4 wk after birth) six male pups from each group were killed for biochemical and hormonal analysis and for measurements of carcass composition. The remaining male animals were maintained on mother's diet for 10 days and then divided in five groups: CC, consisting of offspring born to and suckled by mothers fed a control (C) diet and fed the same diet after weaning; CS, offspring born to and suckled by mothers fed a C diet and fed a soybean flour diet with 17% protein after weaning; LL, offspring of mothers fed a LP diet and fed the same diet after weaning; LC, offspring of mothers fed a LP diet, but fed a C diet after weaning; LS, offspring of mothers fed a LP diet, but fed a soybean flour diet with 17% protein after weaning. The diets are described in Table 1. In the soybean diet, adjustments were made to equalize the carbohydrate, lipids, fiber contents, and energy value to a casein diet, suppressing the soybean oil and reducing fiber. The whole, inactivate soybean flour was obtained by industrial processing (thermal treatment, peeling, grinding, and micronization) that reduced the enzymatic and antitrypsin factor content and contained 80% of the nutritional value of animal casein. Throughout the experimental period, the rats were maintained in individual cages and given free access to food and water. They were kept under standard lighting conditions (12-h light/dark cycle) at a temperature of 24°C. The rats were weight weekly and food intake was daily measured (5 days/wk). The rate of spillage was not accounted for calculation of energy intake. At 90 d of age, the rats were killed by decapitation (between 8:00 and 10:00 AM) and blood samples were collected allowed clotting and the sera stored at -20°C for the subsequent measurement of serum leptin concentrations (Kits Cristal Chem Inc.) and serum insulin concentrations, determined by radioimmunoassay using rat insulin as a standard, ^125^I-labeled bovine insulin as the radioactive tracer, and guinea pig anti-porcine insulin serum as the antibody [24]. The following determinations were performed immediately after decapitation: serum glucose, serum albumin and serum total protein [25-27]. The ratio of serum insulin concentration to serum glucose concentration was calculated to assess the insulin sensitivity. After a medium laparotomy, the retroperitoneal (RET) and perirenal (PERI) white adipose tissues were quickly removed for determination of fresh weight. Carcasses were eviscerated, weighted and stored at -20°C for measurement of their composition.

**Table 1:** Composition of the control, low-protein and soybean flour diets

Ingredient	Control	Low protein	Soybean flour **
	(17% protein)	(6% protein)	(17% protein)
		g/kg	
Casein (84% protein)	202.0	71.5	-
Soybean flour	-	-	415.0
Cornstarch	397.0	480.0	312.2
Dextrinized cornstarch	130.5	159.0	103.7
Sucrose	100.0	121.0	78.6
Soybean oil	70.0	70.0	-
Fiber	50.0	50.0	40.0
Mineral mix (AIN-93)G*	35.0	35.0	35.0
Vitamin mix (AIN-93)G*	10.0	10.0	10.0
L-cystine	3.0	1.0	3.0
Choline bitartrate	2.5	2.5	2.5

*See [Reeves PG, Nielsen FH, Fahey GC Jr: **AIN-93 purified diets for laboratory rodents: final report of the American Institute of Nutrition ad hoc writing committee on the reformulation of the AIN-76A rodent diet**. *J Nutr *1993, **123**:1939–1951.] for more details.

** Composition of soybean flour (100 g): 455 Kcal, 41 g carbohydrate, 25.5 g protein, 21 g total fat, 2 g fiber, 6 g humidity, 4.5 g ash, 100 mg isoflavone.

### Measurements of carcass composition and energy intake

The water carcass was measured by oven drying the carcasses at 80°C until constant weight. Body water was measured by subtracting dry carcass weight from wet carcass weight. The carcass fat was measured after fat extraction in petroleum ether using a Soxhlet continuous fat extractor. Fat content was then calculated by subtracting fat-free dry mass from dry carcass weight. Ash content was estimated following combustion of carcasses at 550°C until constant weight. Protein content was determined by subtracting water, fat and ash content from wet carcass weight.

To calculate energy intake, we assumed the energy content of protein and carbohydrate to be 16.74 Kj/g and fat to be 37.7 Kj/g. The body energy content was calculated from the amount of fat and protein using constant 40 Kj/g fat and 24 Kj/g protein. The calculated baseline energy was estimated by the relationship between carcass components of weaned rats under similar dietary treatment. To CC and CS groups the calculated baseline energy was 793 ± 435 Kj, and to LC, LS and LL groups the value was 188 ± 33 Kj. From the difference between the carcass composition from adult rats and the carcass composition from weaned rats, the lipid gain, protein gain, energy gain, energy efficiency (the ratio of energy gain to energy intake × 100), protein efficiency (the ratio of body weight gain to protein intake), and energy expenditure (the difference between energy intake and energy gain) were calculated.

### Statistical analysis

The results were expressed as mean ± standard deviation for the number of rats (*n*) indicated. The C and LP groups were compared with unpaired t test. Two-way analysis of variance (effects of nutritional status and diet) was used to compare data from CC, CS, LC and LS groups. The same data were analyzed by one-way analysis of variance when assessing whether diet was effective in improving nutritional status in the LC, LS and LL groups. When necessary, these analyses were complemented by the Tukey test to determine the significance of individual differences. Levene's test for homogeneity of variances was initially used to determine whether the data complied with assumptions of parametric analysis of variance. When necessary, the data were log-transformed to correct for variance heterogeneity or non-normality [28]. P < 0.05 indicated significance. All statistical comparisons were done using the Statistic Software package (StatSoft, Inc., Tulsa, OK, USA).

## Results

At birth, rats from dams maintained on low protein diet during pregnancy had body weight lower than that control rats (p < 0.0001). At weaning, the LP group showed hypoalbunaemia (p < 0.002), hypoinsulinaemia and body weight was reduced by 73%, compared to C group (p < 0.001). Serum glucose and leptin concentrations from LP group did not differ from those of the C group (Table 2).

**Table 2:** Body weight at birth and weaning, serum albumin, glucose, insulin and leptin concentrations of weaned rats from mothers maintained on control (C) or low-protein (LP) diets during pregnancy and lactation

	Groups	
	
Variable	C	LP
Body weigth at birth (g)	5.3 ± 0.2 (6)	4.9 ± 0.2* (4)
Body weigth at weaning (g)	103 ± 9 (6)	28 ± 4** (6)
Albumin (g/l)	37 ± 6 (6)	23 ± 5* (6)
Glucose (mmol/l)	5.0 ± 0.9 (6)	5.4 ± 1.2 (6)
Insulin (pmol/l)	387 ± 253 (6)	145 ± 44* (5)
Leptin (pg/ml)	3,598 ± 1,286 (6)	2,759 ± 755 (6)

Values are means ± SD of numbers of litters or rats in parentheses. *Indicates means values significantly different (nonpaired *t *tests; p < 0.05).

Hence, the carcass weight from LP group was also reduced by 72%, compared with that of the C group. Protein restriction interfered significantly with the chemical composition of the carcass: the ash, water, protein and carcass fat content from LP group were, respectively, 2.5, 3.3, 3.7 and 4.7-times lower than for the C group (p < 0.0001). When expressed as a percentage of carcass, the LP group exhibited lipid content 1.3-times lower (p < 0.05) and ash content 1.4-times higher (p < 0.0001) than those in the C group. No difference was found between LP and C groups when the protein and water content were expressed in relation to carcass weight (Table 3).

**Table 3:** Carcass composition of weaned rats from mothers maintained with control (C) or low-protein (LP) diets during pregnancy and lactation

	Groups	
	
Variable	C (6)	LP (6)
	g	
Carcass fresh	75 ± 7	21 ± 4*
Protein	14.0 ± 1.4	3.8 ± 0.4*
Lipid	11.4 ± 0.8	2.4 ± 0.7*
Water	47 ± 5	14 ± 3*
Ash	2.3 ± 0.2	0.9 ± 0.1*
	g/100 g of carcass weight	
Protein	18.8 ± 0.4	18.4 ± 2.1
Lipid	15.4 ± 1.7	11.7 ± 3.0*
Water	62.7 ± 1.4	65.5 ± 3.5
Ash	3.1 ± 0.1	4.4 ± 0.5*

Values are means ± SD for the number of rats in parentheses. *Indicates means values significantly different (nonpaired *t *tests; p < 0.05).

At the beginning of the recovery phase, LC, LS and LL rats had similar body weights and, in all cases, these were significantly lower than those found in CC and CS rats. At the end of this period, body weight of rats from LC and LS groups were lower than those rats from CC and CS groups (F_1,26 _= 173.53, p < 0.0001). Also, rats from CS and LS groups had lower final body weight than those from CC and LC groups (F_1,26 _= 12.73, p < 0.05). Although LS rats had a greater final body weight than LL rats (p < 0.001), their mean weight was still significantly lower than that of the LC rats (p < 0.05). The serum albumin, serum protein, serum glucose and serum leptin concentrations were not significantly among CC, CS, LC and LS rats. Serum insulin was lower in LC and LS groups, as compared to CC and CS groups (F_1,20 _= 48.701, p < 0.0001), but it was higher in CS and LS groups than in CC and LC groups (F_1,20 _= 10.270, p < 0.01). The ratio of serum insulin levels to serum glucose levels was lower in LC and LS groups than in CC and CS groups (F_1,20 _= 26.039, p < 0.0001), and higher in CS and LS groups than in CC and LC groups (F_1,20 _= 8.111, p < 0.01). The In LS and LC rats serum albumin and serum total protein levels were significantly higher than in LL rats (p < 0.01 and p < 0.001, respectively). No difference among these groups was observed in terms of serum glucose and leptin concentrations. Serum insulin and the ratio of serum insulin concentration to glucose concentration were similar in LC and LL rats and significantly lower than LS rats (p < 0.002; p < 0.001, respectively) (Table 4).

**Table 4:** Initial and final body weight, serum total protein, albumin, glucose, insulin and leptin concentrations of adult rats maintained with control (CC and LC) or soybean flour (CS or LS) or low protein (LL) diets after weaning

	Groups				
	
Variable	CC	CS	LC	LS	LL
Initial body weight (g)	182 ± 22 (8)	189 ± 12 (8)	42 ± 11 (7)	45 ± 10 (7)	42 ± 11 (7)
Final body weight (g)	431 ± 27 (8)	387 ± 51 (8)	266 ± 24^a^ (7)	220 ± 25^b^ (7)	114 ± 35^c^ (7)
Total protein (g/L)	63 ± 16 (8)	66 ± 3 (8)	67 ± 3^a^ (7)	64 ± 4^a^ (7)	54 ± 4^b^ (7)
Albumin (g/L)	31 ± 5 (8)	33 ± 4 (8)	29 ± 2^a^ (7)	31 ± 8^a^ (7)	18 ± 8^b^ (7)
Glucose (mmol/L)	3.6 ± 0.9 (6)	3.7 ± 0.5 (6)	3.6 ± 0.9 (6)	3.3 ± 0.8 (6)	3.1 ± 1.2 (5)
Insulin (pmol/L)	170 ± 35 (6)	211 ± 46 (6)	69 ± 18^b^ (6)	117 ± 32^a^ (6)	81 ± 22^b^ (5)
Insulin/glucose ratio	49 ± 12 (6)	58 ± 14 (6)	19 ± 3^b^ (6)	38 ± 15^a^ (6)	28 ± 9^b^ (5)
Leptin (pg/ml)	1,350 ± 371 (6)	1,724 ± 781 (6)	1,012 ± 345 (6)	1,512 ± 623 (6)	1,217 ± 934 (5)

Values are means ± SD for the numbers of rats in parentheses. Means with different superscript letters are significantly different by one-way ANOVA followed by Tukey test (p < 0.05).

Carcass weight, carcass fat and protein contents were lower in LS and LC rats than in CS and CC rats (F_1,26 _= 155.46, p < 0.0001; F_1,26 _= 48.68, p < 0.0001; F_1,26 _= 35.66, p < 0.0001, respectively), as well as in CS and LS groups, compared with CC and LC groups (F_1,26 _= 7.60, p < 0.05; F_1,26 _= 5.87, p < 0.05; and F_1,26 _= 9.23, p < 0.001, respectively). The proportion of fat carcass was reduced only in LC and LS groups when compared with CC and CS groups (F_1,26 _= 15.00, p < 0.001). No difference was observed among groups when the carcass protein content was expressed in relation to body weight. Carcass fresh weight, protein content and the proportion of carcass protein were significantly greater in LS rats, compared to LL rats (p < 0.001), but significantly lower than LC groups (p < 0.05). Carcass lipid content was similar in LS and LL rats, and significantly lower than LC rats (p < 0.05 and p < 0.01, respectively), but the percentage of carcass lipid did not differ among LS, LC and LL rats (Table 5). Absolute and relative RET fat weight was lesser in the LS and LC rats than in the CS and CC rats (F_1,26 _= 114.15, p < 0.0001 and F_1,26 _= 41.52, p < 0.0001, respectively) and in the CS and LS rats, compared to the CC and LC rats (F_1,26 _= 18.96, p < 0.001 and F_1,26 _= 13.84, p < 0.001, respectively). The PERI fat weights of rats from the LS and LC groups were similar, however they were lower than in CS and CC rats (p < 0.01). Concerning body weight, the PERI fat weight was lower in the LS and LC rats than in the CS and CC rats (F_1,26 _= 32.51, p < 0.0001) and in the CS and LS rats, compared to CC and LC rats (F_1,26 _= 12.48, p < 0.01). It was noticed that LS and LL rats had RET fat weights (absolute values) similar between them but significantly lower than LC rats (p < 0.05), whereas PERI fat weights from LS rats were equal to LC and LL rats (p < 0.05). No difference was verified in the relative values of RET and PERI fat weights among three groups of rats (Table 5).

**Table 5:** Carcass composition, absolute and relative weight of white adipose tissue retroperitoneal (RET) and perirenal (PERI) of adult rats maintained with control (CC and LC) or soybean flour (CS or LS) or low protein (LL) diets after weaning

	Groups				
	
Variable	CC (8)	CS (8)	LC (7)	LS (7)	LL (7)
	g				
Carcass fresh	318 ± 42	300 ± 27	199 ± 16^a^	159 ± 186^b^	80 ± 29^c^
Protein	76 ± 5	70 ± 17	50 ± 36^a^	38 ± 4^b^	17 ± 6^c^
Lipid	45 ± 10	36 ± 14	19 ± 86^a^	11 ± 3^b^	8 ± 5^b^
RET	9.93 ± 1.38	7.16 ± 1.57	3.90 ± 1.756^a^	2.18 ± 0.56^b^	1.22 ± 1.10^b^
PERI	3.80 ± 0.73^A^	2.46 ± 0.49^AB^	1.37 ± 0.60^Ca^	0.90 ± 0.36^Cab^	0.43 ± 0.32^b^
	g/100 g of carcass weight				
Protein	24.2 ± 2.9	23.1 ± 5.1	25.0 ± 0.7^a^	24.0 ± 0.4^b^	21.9 ± 0.9^c^
Lipid	14.2 ± 3.0	12.0 ± 5.0	9.6 ± 3.3	6.9 ± 1.0	9.3 ± 3.8
	g/100 g of body weight				
RET	2.30 ± 0.23	1.78 ± 0.34	1.43 ± 0.54	0.98 ± 0.22	0.92 ± 0.61
PERI	0.87 ± 0.13	0.61 ± 0.10	0.50 ± 0.20	0.40 ± 0.13	0.34 ± 0.15

Values are means ± SD for the numbers of rats in parentheses. Means with different superscript capital letters are significantly different by two-way ANOVA and with superscript minuscule letters are significantly different by one-way ANOVA followed by Tukey test (p < 0.05).

**Figure 1:**
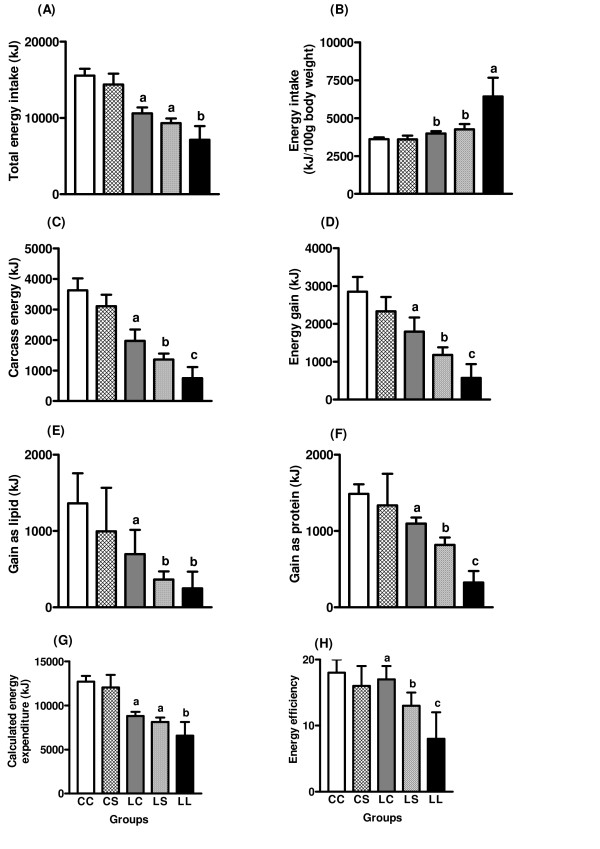
**Total (A) and relative (B) energy intake, carcass energy (C) energy gain (D), gain as lipid (E), gain as protein (F), calculated energy expenditure (G) and energy efficiency (H) of adult rats maintained with control (CC and LC) or soybean flour (CS or LS) or low protein (LL) diets after weaning**. Values are means ± SD for the numbers of rats in parentheses. Means with different superscript letters are significantly different by one-way ANOVA followed by Tukey test (p < 0.05).

The total energy intake during the recovery phase was lower in LC and LS rats, compared to CC and CS rats (F_1,26 _= 188.99, p < 0.0001) and in CS and LS groups than in the CC and LC groups (F_1,26 _= 11.14, p < 0.01). During the recovery phase, the total energy intake was similar in the LS and LC groups and significantly greater than in the LL group (Figure 1A). When expressed per gram of body weight, the energy intake was higher in the LC and LS groups, in relation to the CC and CS groups (F_1,26 _= 37.58, p < 0.0001) (CC = 3609 ± 120 Kj/100 g body weight, CS = 3593 ± 251 Kj/100 g body weight, LC = 3987 ± 154 Kj/100 g body weight, LS = 4264 ± 350 Kj/100 g body weight). Rats from LS and LC groups had energy intake/100 g body weight similar among them, but significantly lower than the LL group (6432 ± 1230 Kj/100 g body weight, p < 0.001) (Figure 1B).

Also, rats from LC and LS groups had smaller carcass energy (F_1,26 _= 178.68, p < 0.0001), energy gain (F_1,26 _= 74.89, p < 0.0001), gain as lipid (F_1,26 _= 20.12, p < 0.0001), gain as protein (F_1,26 _= 27.31, p < 0.0001), calculated energy expenditure (F_1,26 _= 149.34, p < 0.0001) and energy efficiency (F_1,26 _= 8.36, p < 0.01) than rats from CC and CS groups. These variables were lower in the CS and LS groups than in the CC and CS groups (F_1,26 _= 19.77, p < 0.0001, F_1,26 _= 19.77, p < 0.001, F_1,26 _= 5.87, p < 0.03, F_1,26 _= 6.42, p < 0.02, F_1,26 _= 4.35, p < 0.05 and F_1,26 _= 11.77, p < 0.01, respectively) (Figures 1C–1H). Carcass energy (Figure 1C), energy gain (Figure 1D), gain as protein (Figure 1F) and energy efficiency (Figure 1H) were lower in the LS group than in LC group (p < 0.05), but higher than in the LL group (p < 0.05). The gain as lipid (Figure 1E) was similar in the LS and LL groups but significantly lower than in the LC group (p < 0.05). In the LS and LC groups, the calculated energy expenditure (Figure 1G) did not differ and was significantly higher than LL group (p < 0.05).

## Discussion

In this study, protein restriction during fetal and neonatal life led to a low weight at birth and at weaning, in agreement with our previous observations [29]. At weaning, the poor nutrition status was confirmed by hipoalbuminemia and hipoinsulinemia, features commonly found in malnourished infants and in animals [30,31]. In contrast to what has been observed in those subjected to protein restriction during growth phase after weaning which showed increases in the proportion of body fat, in this study we verified decreases in the total fat mass and percentage of body fat, but no alteration in the proportion of body protein [11,32]. Thus, it seems that protein restriction in early life affects body fat to a greater degree than it does body protein.

Undernourished rats and those subjected to protein-restricted during intrauterine life show similar and higher body weights, respectively, after nutritional recovery, compared to control rats [13,15,33]. In this and in previous studies, nutritional rehabilitation after weaning did not correct the body weight deficit, regardless of dietary protein quality [34,35]. Despite low body weight, the proportion of protein carcass remained similar in recovered and control rats, in contrast to study showing that rats submitted to prenatal and early postnatal protein malnutrition present reduced fat-free dry mass protein after nutritional rehabilitation [36]. Based on these observations, prenatal period and caloric restriction are more critical for obesity genesis than the postnatal period and protein restriction.

Obesity is thought to be result of an energy intake – energy expenditure imbalance. Although both recovered groups have showed higher energetic intake relative to body weight and lower energy expenditure, they exhibited lower proportion of carcass fat and fatty deposits than control rats, an indication that our model did not induce obesity. However, this study ended when the rats were 90 days old and it is possible that body composition changes as rats grow old.

Soybean diet has been associated to reduction in the fat deposits due to the action of isoflavone that increases energy expenditure by altering the brown-fat activity and the thyroid function [21]. In this study, soybean diet was more effective than the casein diet in reducing the proportion of fat deposits, although the animals maintained on soybean diet had eaten proportionally the same amount of diet and had showed lower energy expenditure than those fed the casein diet. It has been reported that genistein, an isoflavone of soy, decreases lipogenesis, enhances lipolysis and counteracts the antilipolytic action of insulin in isolated rat adipocytes genistein [22,37]. Recently, we reported that our rats fed with the soybean diet show considerable amount of genistein in the sera [38] and express less mRNA of acetyl-CoA carboxylase in the liver [39]. Thus, the reduced adiposity seen in our animals maintained on soybean diet could be an effect of this isoflavone on lipid metabolism.

However, we observed a lower energy efficiency of soybean diet than casein diet. Especially in the LS group, the low energy efficiency was reflected in less energy gain as protein and proportion of carcass protein, and lower energy gain as lipid. We have recently verified that our soybean diet has lower digestibility than the casein diet (RV Veloso, MQ Latorraca, VC Arantes, MAB Reis, F Ferreira, AC Boschero and EM Carneiro unpublished results), and perhaps this effect was critical to accretion of carcass protein in animals that suffered previous nutritional deficit. Nevertheless, since the rats maintained with the soybean or casein diet showed serum albumin and total protein concentrations similar to control rats and higher as compared to malnourished rats, it became clear that the soybean diet was efficient for the recovery of the nutritional status of the rats. The lower accretion of lipid in depots and in carcass in animals recovered with soybean diet could be beneficial if in our animal model age determine changes in the body composition, favoring the development of obesity.

In agreement to what has been observed by others [40,41], we verified that basal concentration of serum leptin did not differ among weaned protein-deprived rats, in the adults rats recovered from early protein restriction and in the control rats. Although isoflavones have been associated with reduction in leptin levels [18,21], our rats fed with the soybean diet had similar leptin levels to those fed the casein diet. It is known that insulin and glucose together regulate leptin production and secretion [42]. Moreover, genistein increases insulin secretion by isolated islets in the presence of basal, physiologic and supraphysiologic glucose concentrations [43,44]. In this study, rats kept on a soybean diet showed high serum insulin concentrations, as compared with those fed with a casein diet. In contrast, recovered and malnourished rats showed reduction in the serum insulin concentrations, as previously described [34]. Equivalent serum leptin levels in rats exhibiting reduced proportion of fat depots and low insulin/glucose ratio, such as it was seen in our LC and LL groups in relation to CC and CS, are consistent with the concept that early protein restriction enhances the leptin response, by increasing insulin sensitivity of adipocyte glucose uptake [41]. On the other hand, in rats maintained on soybean diet, the same effect was perhaps favored by high insulin levels, which appeared to stimulate leptin production [45].

Circulating levels of leptin are proportional to the total fat mass, and food intake and energy expenditure are regulated by leptin [46,47]. In this study, recovered and malnourished rats, as well as those fed with soybean diet that exhibited lower fat mass, had similar serum leptin levels when compared to control rats and animals fed with casein diet. This suggests that although these animals possess fewer adipocytes, they obtain a higher amount of leptin than their controls. Also, we observed that energy expenditure appear to be related to body weight. Interestingly, serum leptin concentrations were not associated to energy expenditure or to energy intake. Even so, maintenance of normal serum leptin concentrations can be beneficial to prevent leptin resistance and obesity.

Thus, our results indicate that protein restriction during intrauterine life and lactation periods did not provoke obesity in adulthood, despite the increase in food intake in relation to body weight and reduction of energy expenditure. Nutritional recovery with soybean diet did not interfere in the serum leptin concentration, food behavior and energy expenditure, but decreased the body weight at the expense of lower energy efficiency with repercussion on lean mass.

## Competing interests

The authors declare that they have no competing interests.

## Authors' contributions

LMGC and EAD carried out the measurements of carcass composition and energy intake. VCA carried out the RIA. RVV, MHGGS, MABR, EMC and ACB contributed equally to this paper in various aspects of this study. MQL conceived of the study and designed this study. LMGC drafted the manuscript along with the other authors. All authors read and approved the final manuscript.
